# LB10. Impact of SARS-CoV-2 Delta Variant on the Spectrum of Pediatric COVID-19 Disease in Arkansas

**DOI:** 10.1093/ofid/ofab466.1641

**Published:** 2021-12-04

**Authors:** Jose R Romero, Donald E Warden, Michael Cima

**Affiliations:** 1 University of Arkansas for Medical Sciences, Little Rock, Arkansas; 2 Arkansas Department of Health, Little Rock, Arkansas

## Abstract

**Background:**

Pediatric SARS-CoV-2 infection is generally thought to be asymptomatic or result in mild COVID-19 disease, with a paucity of severe outcomes. However, SARS-CoV-2 variants, notably B.1.617.2 (WHO Delta), have changed the clinical landscape of COVID-19 in the United States. Delta became the dominant variant in Arkansas (AR) the 1^st^ week of July 2021. Schools contributed to pediatric infections during the January 2021 surge in COVID-19 infections even with physical mitigation measures (PMM) that were removed in March 2021. We present preliminary data suggesting a shift in the clinical presentation of children with Delta variant infection.

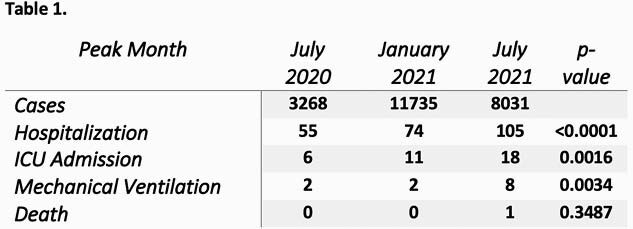

**Methods:**

Pediatric (ages ≤ 18 years) case records for the 3 months representing key inflection points of the COVID-19 Pandemic in AR were reviewed. Outcomes (hospitalizations, ICU admission, mechanical ventilation, death) were recorded by the Arkansas Department of Health (ADH) in a statewide database. Fisher’s Exact Test was used with p-values < 0.05 indicating statistical significance.

**Results:**

During July 2020, 3,268 pediatric cases were reported to ADH with 55 hospitalizations, 6 ICU admissions, 2 mechanical ventilations, and no deaths. A second peak in January 2021 included 11,735 pediatric cases, a 259.1% increase. Increases were also seen in hospitalizations (n=74), ICU admissions (n= 11), and mechanical ventilations (n=2). No deaths reported. The beginning of an exponential growth in cases during July 2021, before the opening of schools, included 8,031 pediatric cases. Despite 31.6% fewer cases than the previous peak, hospitalizations increased 41.9% (n=105) (p < 0.0001) and included increases in ICU and ventilator use of 68.6% (n=18) (p 0.0016) and 300% (n=8) (p 0.0034), respectively. One pediatric death was reported. (Tbl 1)

**Conclusion:**

In the absence of PMM and despite the summer closure of schools, pediatric COVID-19 cases and severe outcomes increased significantly. Initial analysis of the AR July 2021 Delta variant surge indicates a statistically significant increase in pediatric COVID-19 disease and severity as indicated by a proportional increase in hospitalizations, ICU, and ventilator use. Further studies are warranted to better define Delta related childhood disease. Our findings also have implications for school PMM efforts.

**Disclosures:**

**All Authors**: No reported disclosures

